# Effect of Synergistic Action of Bovine Lactoferrin with Antibiotics on Drug Resistant Bacterial Pathogens

**DOI:** 10.3390/medicina57040343

**Published:** 2021-04-02

**Authors:** Mohammed S. Al-Mogbel, Godfred A. Menezes, Mohamed T. Elabbasy, Manal M. Alkhulaifi, Ashfaque Hossain, Mushtaq A. Khan

**Affiliations:** 1 Clinical Laboratory Sciences Department, College of Applied Medical Sciences, Ha’il University, Ha’il P.O. Box 2240, Saudi Arabia; msm_hhscb@hotmail.com; 2 Department of Medical Microbiology & Immunology, RAK College of Medical Sciences (RAKCOMS), Central Research Laboratory (CRL), RAK Medical & Health Sciences University (RAKMHSU), Ras al Khaimah P.O. Box 11172, United Arab Emirates; ashfaque@rakmhsu.ac.ae; 3 College of Public Health and Centre for Molecular Diagnostics and Personalized Therapeutics (CMDxPT), Ha’il University, Ha’il P.O. Box 2240, Saudi Arabia; tharwat330@gmail.com; 4 Department of Botany & Microbiology, College of Science, King Saud University, Riyadh P.O. Box 11451, Saudi Arabia; manalk@ksu.edu.sa; 5 Department of Microbiology and Immunology, College of Medicine and Health Sciences, United Arab Emirates University, Al Ain P.O. Box 15551, United Arab Emirates; mushtaq.khan@uaeu.ac.ae

**Keywords:** antimicrobial resistance, synergism, bacteria, lactoferrin

## Abstract

*Background and Objectives*: The multidrug resistant (MDR) bacterial pathogenic infection is one of the chief worldwide public health threat to humanity. The development of novel antibiotics against MDR Gram negative bacteria has reduced over the last half century. Research is in progress regarding the treatment strategies that could be engaged in combination with antibiotics to extend the duration of these life-saving antibacterial agents. The current study was therefore planned to assess the synergistic effects of bovine lactoferrin (bLF) in combination with different antibiotics that are conventionally used. This synergism would provide a newer therapeutic choice against MDR pathogens. LF is present in mucosal secretions, vastly in milk. LF is considered an important constituent in host defense. In previous reports, LF has been co-administered as a combination antibiotic therapy. *Materials and Methods*: This study included synergistic (LF + appropriate antibiotic) exposure against 147 locally encountered bacterial pathogens, which were completely characterized strains. The anti-biofilm effects and the outcome of bLF on minimum inhibitory concentrations (MICs) of antibacterials on clinical MDR bacterial pathogens were determined by standard techniques. *Results*: In our study, synergism of bLF with antibacterial agents were reproducible and found to be significant. LF on its own had an important effect of inhibiting the biofilm production of some significant bacterial pathogens. *Conclusion*: The results of this study provides useful data on the antibacterial potential of the combination of LF with antibiotics against drug resistant pathogens.

## 1. Introduction

Lactoferrin (LF) is a constituent of the innate immune system found in human and animal mucosal secretions has been postulated to take part in a potential therapeutic role, including: a. Increasing antimicrobial susceptibility to particular antibiotics and b. Preventing biofilm formation. Further, dearth in production of innate lactoferrin appears to affect some individuals to enhanced risk of infection [1].

LF belongs to transferrin family and it is a non-heme iron binding protein [2]. It is present in different secretions, like saliva, tears, nasal and bronchial secretions and most vastly in milk [3]. These fluids line with the body’s external environment and take part in a significant role in the innate immune system. LF is present in substantial amounts in neutrophil and it takes an important role in reducing pathogen level [4]. Hence, it is considered important constituent in the first line of host defense [5]. LFs binding has been detected at concentrations down to 1 mg/mL, which is the concentration in normal serum [6]. It is released in significant quantities in the course of inflammation [7]. 

The clinical studies have put forward LF as a potential prophylactic agent for a number of infections [8]. The LF has activity (both in vitro and in vivo) on a huge variety of pathogens [9]. Further, scientists have tested varied doses against extensively different strains of bacteria or fungus. Overall, many beneficial property for the intact proteins as well as some of its peptides have been reported. Some of these seem to have “direct” antimicrobial effects that can be measured using easy minimal inhibitory concentration (MIC) testing on cultures. The antibacterial power of LF against all the microbes has been explored. LF also has modulatory effect on inflammatory response [10]. LF chelates iron, in turn making this critical ion unavailable to the attacking microbes [11]. 

Scientific studies have shown that LF can act as either a bacteriostatic and/or bactericidal agent [10]. The difference in the activity explains the wide range of MIC values for LF. Presence of LF receptors on the surface of these microorganisms may partially explain the resistance of isolates to LF. The result of LF as a co-administered adjuvant therapy in Gram-negative antibiotic treatment has been published previously [12]. The aim of this study was to determine the effect of bovine lactoferrin (bLF) on MICs of important antibiotics against drug resistant clinical bacterial pathogens cultured in the region of Ha’il, KSA. The results generated out of this study would certainly help in the adjuvant based treatment methods for locally faced antimicrobial resistant pathogens. 

## 2. Materials and Methods

This study included synergistic (LF + appropriate antibiotic) exposure against a total of 147 locally encountered bacterial pathogens, including completely characterized strains, including Methicillin resistant *Staphylococcus aureus* (MRSA); Vancomycin resistant Enterococci (VRE); Extended-spectrum β-lactamase (ESBL) producing *Enterobacteriaceae*; Fluoroquinolone resistant *Salmonella* spp.; AmpC β-lactamase producing *Enterobacteriaceae*; Carbapenem resistant *Enterobacteriaceae*; multidrug resistant (MDR) *Pseudomonas* & *Acinetobacter* species; *Streptococcus mutans* and *Lactobacillus* spp. cultured from patients attending the medical facilities in Ha’il and surrounding regions and also the non-typhoidal *Salmonella* isolates out of the food poisoning cases in the Ha’il region and the surrounding regions. The commercially available lactoferrin derived from bovine milk was obtained.

### Isolation & Identification of Bacterial Pathogens

Identification of bacterial strains were carried out by using manual methods including catalase, coagulase and mannitol fermentation tests, and Gram-staining. Further the identification were be confirmed by and Matrix-assisted laser desorption/ionization-time of flight mass spectrometry (MALDI-TOF-MS) (Bruker Daltonik GmbH, Bremen, Germany) and Microscan (Beckman Coulter, Brea, CA, USA), according to the manufacturer’s guidelines [13,14]. A bacterial colony was placed in duplicate on a MALDI-TOF-MS plate and the results were noted. 

*Susceptibility testing of bacterial pathogens:* Kirby Bauer disk diffusion method & also Microscan method were carried out. For Kirby Bauer method following antibiotic discs for Gram positives: ampicillin (10 μg), amoxicillin (30 μg), cefepime (30 μg), cefotaxime (30 μg), cefuroxime (30 μg), cephalexin (30 μg), ciprofloxacin (5 μg), chloramphenicol (30 μg), clindamycin (2 μg), erythromycin (15 μg), gentamicin (10 μg), methicillin (5 μg), rifampicin (5 μg), oxacillin (1 μg), streptomycin (10 μg), roxithromycin (15 μg), trimethoprim-sulfamethoxazole (5 μg) and vancomycin (30 μg). For Gram negatives the antimicrobials tested were as follows, cotrimoxazole (25 µg), nalidixic acid (10 µg), ciprofloxacin (5 µg), chloramphenicol (30 µg), ampicillin (10 µg), ceftazidime (30 µg), ceftriaxone (30 µg), colistin (10 μg), meropenem (10 µg), tigecycline (15 μg) and polymixin (300 units). MIC values were determined following standrad interpretative standards. The selected isolates were preserved by lyophilization and freezing at −80 °C for further use.

*Phenotypic testing for ESBLs:* Strains resistant to 3rd generation cephalosporins (one or more) were considered screen positive for ESBLs and were confirmed by the combination disk method. IsolateS were tested for ESBL by the combination disk method using ceplaosporin versus cephalosporin + beta-lactamase inhibitor, comprising ceftazidime (30 µg); cefotaxime (30 µg); ceftazidime-plus-clavulanate (30 µg plus 10 µg) and cefotaxime-plus-clavulanate (30 µg plus 10 µg). A ≥5 mm increase in diameter of the inhibition zone of the cephalosporin + beta-lactamase inhibitor disc, when compared to the respective cephalosporin disc alone were interpreted as phenotypic evidence of ESBL production. 

*Phenotypic testing for AmpC β-lactamases:* Strains resistant to Cefoxitin were suspected to be the AmpC β-lactamases producers. Confirmation of AmpC β-lactamases was directly using the bacterial isolates. It is a technical variation of the conventional three dimensional extract test: In this test, a heavy inoculum was streaked over the agar surface in a linear fashion, beginning 5 mm from the disc and moving outwards. Cefoxitin discs were placed centrally on the plates and overnight incubation at 37 °C was carried out [15]. 

*Inducibility (Chromosome mediated) of AmpC β-lactamases*: It was detected by Disk Antagonism Test (DAT). Disks of inducing agent cefoxitin (Cn) and cephalosporins (Cpm, Ca, Ci and Ce) were placed on the surface of the test bacterial lawn on MHA plates. The plates were examined after overnight incubation at 37 °C. Imipenem was used as an inducing agent and compared with cefoxitin, in the disk antagonism test [16].

*Plasmid-mediated (derepressed, transferable) AmpC β -lactamases:* These were detected by AmpC disk test. Lawn culture of *E. coli* ATCC 25922 was prepared on MHA. Sterile disk (6 mm) were moistened with sterile saline (20 µL) and inoculated with several colonies of test organism. The inoculated disk were then placed beside a cefoxitin disk (almost touching) on the inoculated plate.

*Phenotypic detection of carbapenemase production:* (a) Modified Hodge’s test (MHT): All the isolates meropenem resistant isolates were screened for metallo-β-lactamase (MBL) and *Klebsiella pneumoniae* carbapenemase (KPC) production by modified Hodge’s test (MHT) [17]. (b) Imipenem-I EDTA (I-EDTA) synergy test: All the imipenem resistant isolates were tested by imipenem-I EDTA (I-EDTA) synergy test for MBL production [17].

*Preparation of template DNA:* The DNA of the bacterial strains were isolated by using Qiacube using the Qiagen DNA isolating kits (Qiagen, Hilden, Germany), according to the manufacturer’s guidelines.

*Detection of genes responsible for MRSA and VRE:* Sequences of primers used for MRSA detection were as per Felmingham et al., 2002 [18]. The primers used for VRE detection were as per Miele et al., 1995 [19].

*Molecular analysis of quinolone resistance:* The mechanism of quinolone resistance were determined by investigating mutations in the DNA gyrase (*gyrA* and *gyrB*) and DNA topoisomerase IV (*parC* and *parE*) genes as per Menezes et al., 2012 [20].

*Sequence analysis of gyrA, gyrB, parC and parE polymerase chain reaction (PCR) products:* Sequencing were performed with both forward and reverse primers (same as used for the PCR) on Eppendorf DNA Thermal Cycler and analyzed in an automatic DNA sequencer 3130X1 Genetic Analyzer (ABI PRISM) AB (Foster City, CA, USA). DNA sequences were analyzed by using a commercial software (Lasergene; DNAStar, Inc., Madison, WI, USA). The BLASTN program were used for database searching (http://www.ncbi.nlm.nih.gov/BLAST/).

*β-lactamase detection: PCR screening & sequence analysis:* Isolates resistant to extended-spectrum cephalosporins were initially screened for the presence of TEM and SHV β-lactamases using Check-Points BV. PCR screening and sequencing (Sanger sequencing) of the extended-spectrum cephalosporin resistant isolates were performed to identify the β-lactamase resistance genes; *bla*_TEM_, *bla*_SHV_, *bla*_OXA-1_ group, *bla*_CTX-M_ and AmpC. Sequencing was performed using both forward and reverse PCR primers and standard methods on a 3130X1 Genetic Analyzer (ABI PRISM). The BLASTN program was used for database searching (http://www.ncbi.nlm.nih.gov/BLAST/). Additional sequencing primers were required for *bla*_TEM_ PCR product sequencing (Lagging strand 7, 5′-TTACTGTCATGCCATCC-3′ and Lagging strand 3, 5′-AGAGAATTATGCAGTGC-3′). PCR primers corresponding to sequences downstream (ORF 1) of the *bla*_CTX-M_ genes (M3 int upp, 5′-TCACCCAGCCTCAACCTAAG-3′ and ORF1 pol M3, 5′-GCACCGACACCCTCACACCT-3′ were also used.61 Finally, PCR products of *bla*_CTX-M_ were subjected to sequencing using primers, CTX-M-1 fw multi 5′-AAAAATCACTGCGCCAGTTC-3′, CTX-M-1 multi (REV)F seq 5′-AACGTGGCGATGAATAAGCT-3′ and ORF1 pol M3, 5′-GCACCGACACCCTCACACCT-3′ [20]. 

A multiplex PCR for the simultaneous detection of the carbapenemase genes using primers targeting *bla*_KPC_, *bla*_NDM-1_, *bla*_IMP_ and *bla*_VIM_ gene was done. The primers used for the amplification by multiplex PCR for the simultaneous detection of the carbapenemase genes were as per Mulvey et al., 2011 [21].

*The antimicrobial agents for synergism testing with lactoferrin:* The dialysis method of reconstitution of commercial LF was successfully optimized and employed [10 mg/mL = 0.01 g/mL = 0.05 g/5 mL—dissolved in ultrapure sterile water. After dialysis (against 0.2 M sodium acetate) the volume was doubled with the volume with ultrapure sterile water. So the final concentration is = 0.5 mg/mL].

*Preparation of LF:* The bLF were purchased from Sigma Chemical Company (St. Louis, MO, USA). Dissolved in dionized water in appropriate concentration. Iron-free lactoferrin were prepared by dialysis against–0.2 M sodium acetate–0.2 M NaH_2_PO_4_–0.4 M EDTA, pH = 4.0.

The dialysis tubes were removed from storage distilled water, then were knotted carefully at one end, filled with each of LF and knotted carefully at other end. Immersed dialysis tubes in a beaker or flask, and dialysed against above solution for 16 h with gentle stirring at temperature of 4 °C and changing solution every 4 h, then the tube is transferred into dionized water for 4 h prior to use. The dialysis tubes from the buffer were removed. The membrane was held vertically, the excess buffer was removed and sample was removed with a pasteur pipet [22]. 

Preliminary procedure for testing the effect of bLF alone and with the synergism accompanied by an antibiotic compound: For the preliminary testing protocol, Kirby Bauer disk diffusion testing was employed to test the effect of lactoferrin alone and with the synergism accompanied by an antibiotic compound. 

Study of synergistic action of LF on drug resistant bacterial pathogens using MicroScan WalkAway (Beckman Coulter, Brea, CA, USA), an automated bacterial identification and MIC based susceptibility testing system: The 10 µL of the reconstituted 0.5 mg/mL of the LF was added to 90 µL of the bacterial suspension in the Microscan Walkaway system panel for susceptibility testing. 

The protocol was optimized and tested for reproducibility and used for the test. The study included synergistic (LF + appropriate antibiotic) exposure against a total of 147 locally encountered, completely characterized strains cultured from patients attending the hospitals in Ha’il and surrounding regions.

*Molecular Biology based evaluation of the study:* The molecular biology based study for the genes responsible for resistance against particular class of antibiotics was performed before and after the synergistic exposure to LF. There was no observable change in the molecular basis (genes coding for broad-spectrum β-lactamases) of drug resistance.

*Lactoferrin inhibition of biofilm production:* The effect of natural LF on the ability of biofilm formation of pathogens was studied using a microculture protocol against following pathogens—ESBL producing clinical *Escherichia coli*; Carbapenemase producing (cephalosporin and carbapenem resistant) and ciprofloxacin resistant *Citrobacter freundii*; ESBL producing clinical *Enterobacter aerogenes*; Carbapenemase producing (cephalosporin and carbapenem resistant) clinical *Enterobacter aerogenes*; Carbapenemase producing (cephalosporin and carbapenem resistant) clinical *Pseudomonas aeruginosa*; Methicillin resistant *Staphylococcus epidermidis* (MRSE); Methicillin resistant *Staphylococcus aureus* (MRSA) and Vancomycin resistant *Enterococcus faecium* (VRE). 

## 3. Results

**Figure 1:**
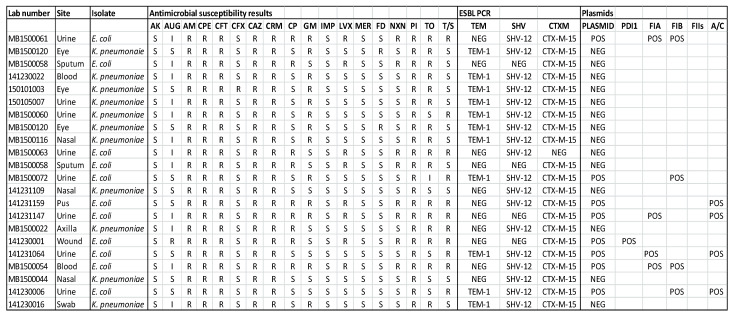
Antimicrobial susceptibility pattern; ESBL PCR results and plasmids found in the representative clinical *Enterobacteriaceae* isolates. Note: S—Susceptible; I—Intermediate; R—Resistant. Site—site of sampling in patients. Antimicrobial agents listed: AK—amikacin, AUG—augmentin, AM—ampicillin, CPE—cefepime, CFT—cefotaxime, CFX—cefoxitin, CAZ—ceftazidime, CRM—cefuroxime, CP—ciprofloxacin, GM—gentamicin, IMP—imipenem, LVX—levofloxacin, MER—meropenem, FD—nitrofurantoin, NXN—norfloxacin, PI—piperacillin, TO—tobramycin, T/S—cotrimoxazole.

**Figure 2:**
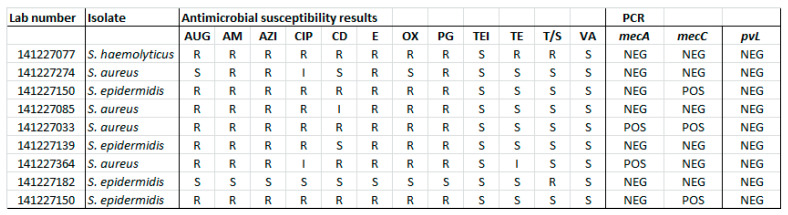
Antimicrobial susceptibility pattern; PCR results for *mecA*, *mecC* and *pvL* genes among representative *Staphylococcus* species. Note: S—Susceptible; I—Intermediate; R—Resistant. Antimicrobial agents listed: AUG—augmentin, AM—ampicillin, AZI—azithromycin, CP—ciprofloxacin, CD—clindamycin, E—erythromycin, OX—oxacillin, PG—penicillin, TEI—teicoplanin, TE—tetracycline, T/S—cotrimoxazole, VA—vancomycin.

Antimicrobial susceptibility pattern; PCR results among representative isolates are shown in the Figure 1 and Figure 2. For the preliminary testing protocol, by Kirby Bauer disk diffusion testing LF alone (non-dialyzed or dialyzed) or along with gentamicin with different concentrations (0.5 mg/mL; 10 mg/mL—both 5 µL) did not make any difference to zone of inhibition when compared with gentamicin alone. The effect of LF was also tried to be tested by the well inoculum method, using 5 µL of LF. Hence the effect of LF was not demonstrable by the disk diffusion method or well inoculum method.

**Figure 3:**
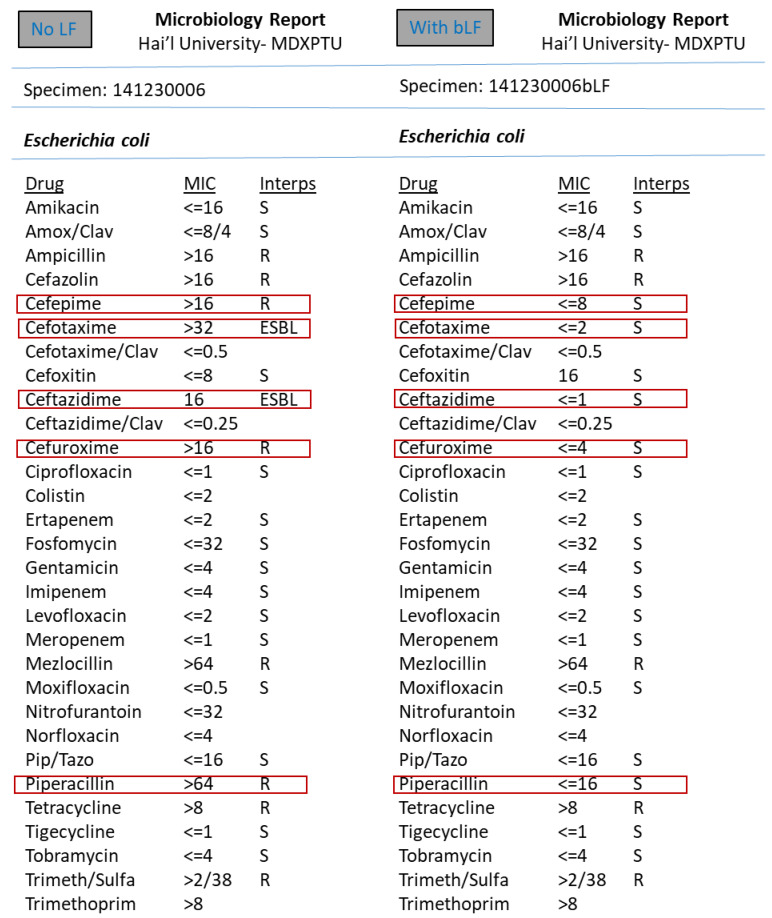
Demonstration of effect of bovine lactoferrin (bLF) on Extended spectrum β-lactamase producing clinical *Escherichia coli.*

**Figure 4:**
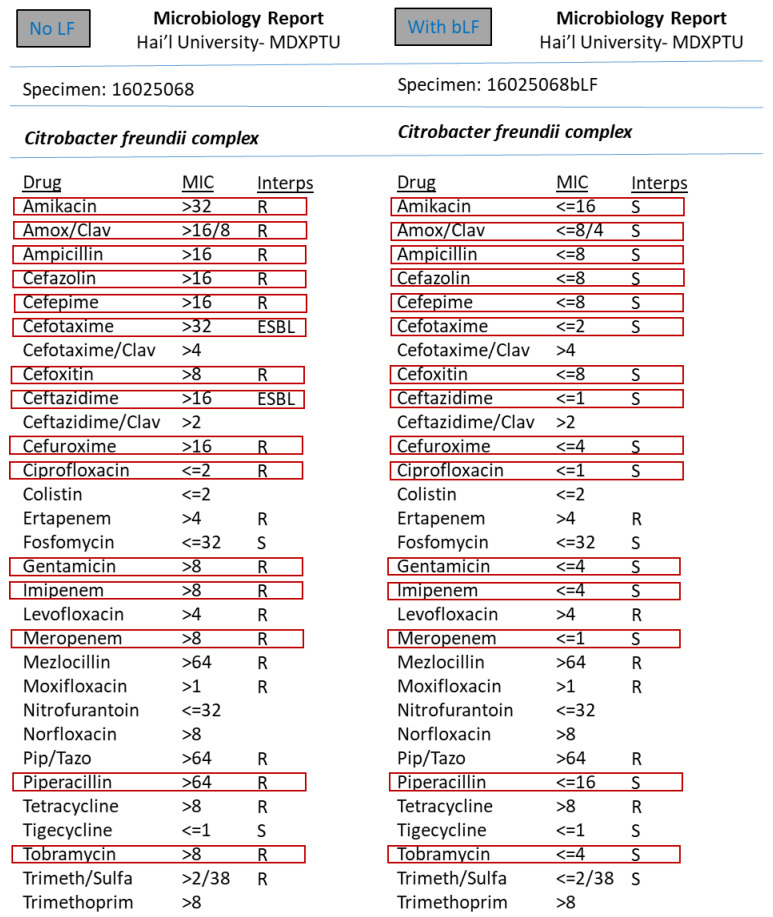
Demonstration of effect bovine lactoferrin (bLF) on Carbapenemase producing (cephalosporin and carbapenem resistant) and ciprofloxacin resistant *Citrobacter freundii.*

**Figure 5:**
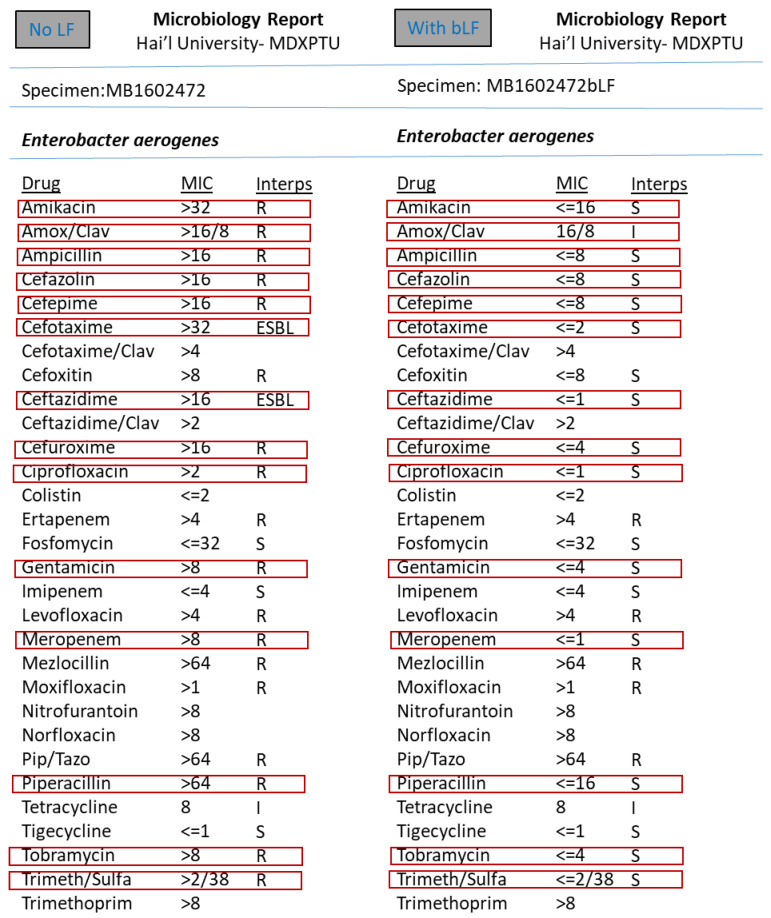
Demonstration of effect of bovine lactoferrin (bLF) on Carbapenemase producing (cephalosporin and carbapenem resistant) clinical *Enterobacter aerogenes.*

**Figure 6:**
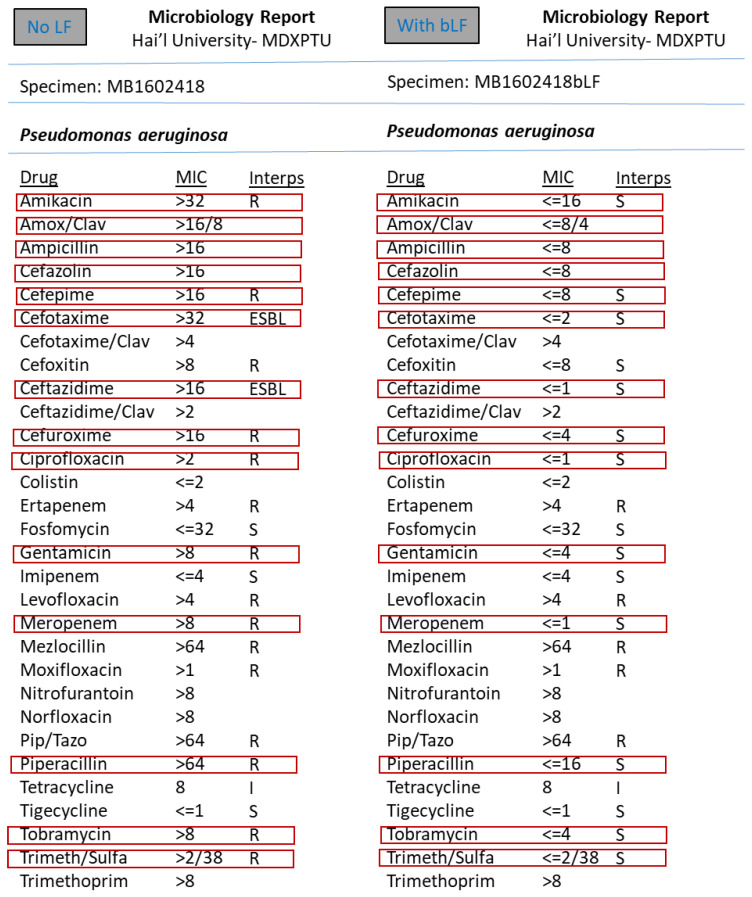
Demonstration of effect of bovine lactoferrin (bLF) on Carbapenemase producing (cephalosporin and carbapenem resistant) clinical *Pseudomonas aeruginosa.*

**Figure 7:**
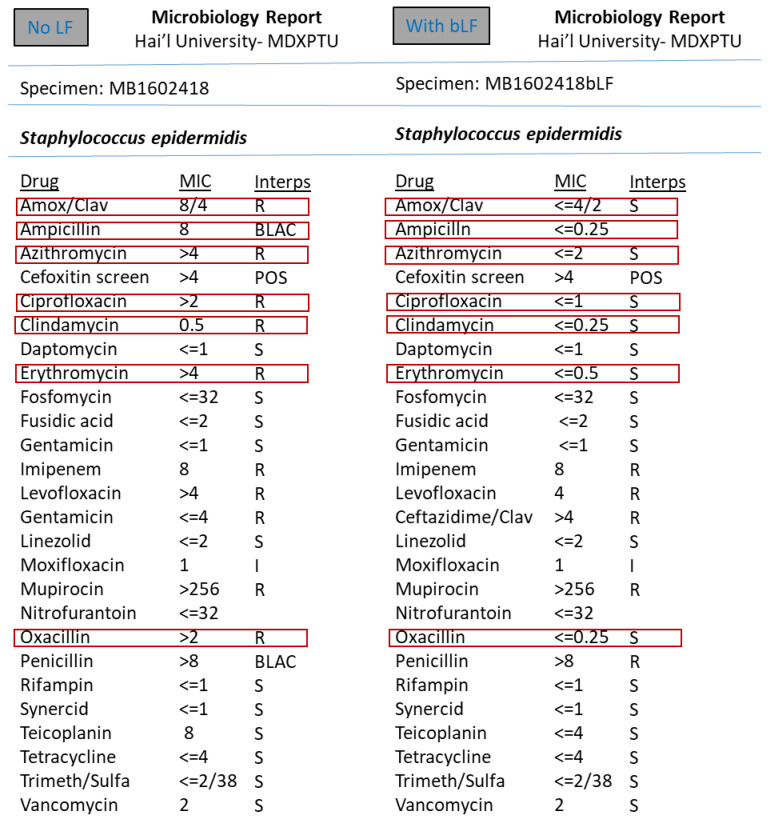
Demonstration of effect of bovine lactoferrin (bLF) on Methicillin resistant *Staphylococcus epidermidis* (MRSE).

**Figure 8:**
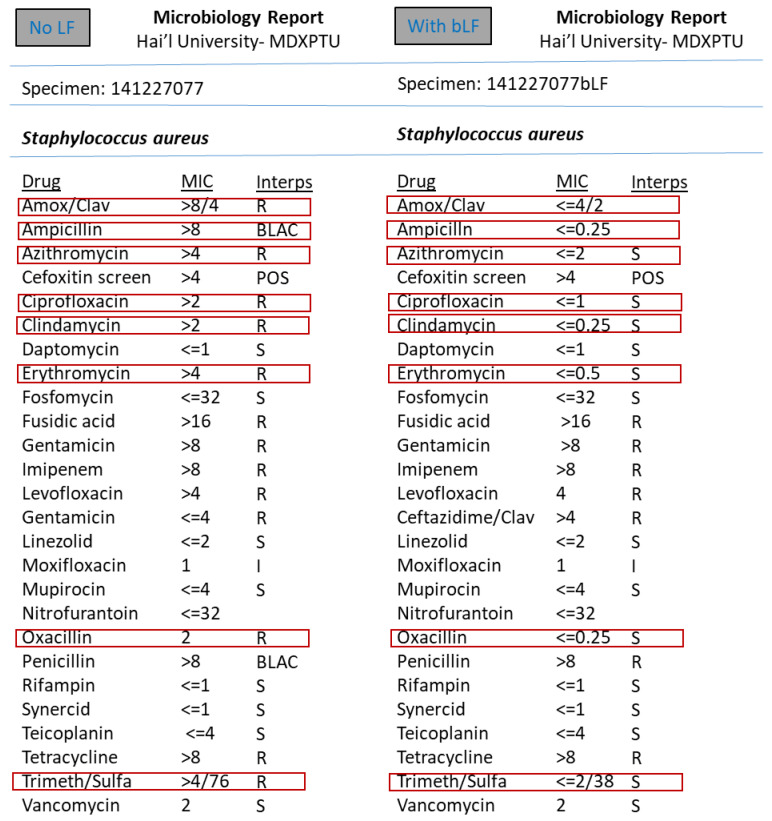
Demonstration of effect of bovine lactoferrin (bLF) on Methicillin resistant *Staphylococcus aureus* (MRSA).

**Figure 9:**
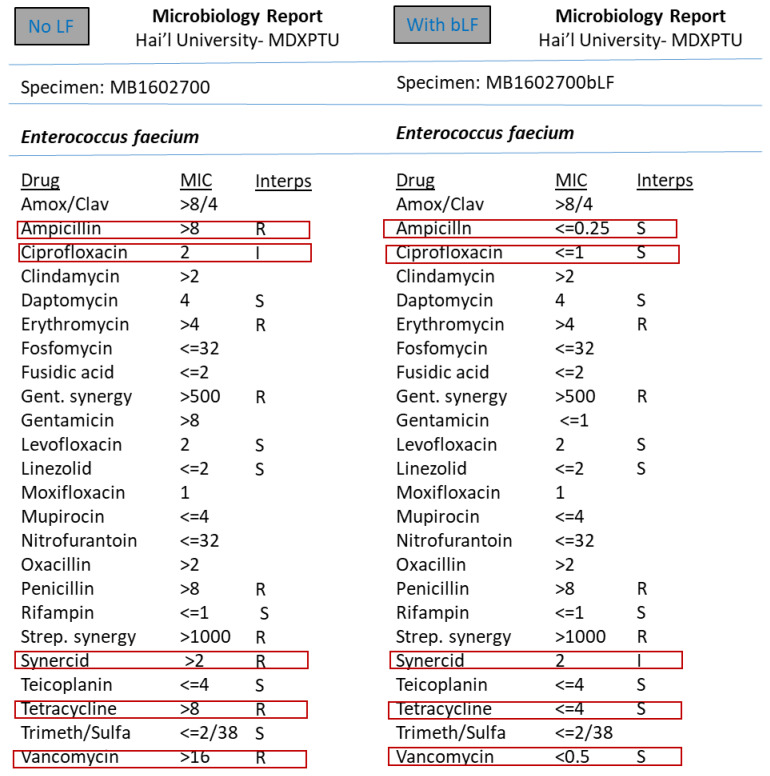
Demonstration of effect of bovine lactoferrin (bLF) on Vancomycin resistant *Enterococcus faecium* (VRE).

However, the synergism effect of LF with antimicrobial compounds (antibiotics) after optimising the MIC (minimum inhibitory concentration) based tests were found to be remarkable. Figure 3, Figure 4, Figure 5, Figure 6, Figure 7, Figure 8 and Figure 9 demonstrates effect of bLF on ESBL producing clinical *Escherichia coli*; demonstration of effect of bLF on Carbapenemase producing (cephalosporin and carbapenem resistant) and ciprofloxacin resistant *Citrobacter freundii*; demonstration of effect of bLF on Carbapenemase producing (cephalosporin and carbapenem resistant) clinical *Enterobacter aerogenes*; demonstration of effect of bLF on Carbapenemase producing (cephalosporin and carbapenem resistant) clinical *Pseudomonas aeruginosa*; demonstration of effect of bLF on Methicillin resistant *Staphylococcus epidermidis* (MRSE); demonstration of effect of bLF on Methicillin resistant Staphylococcus aureus (MRSA) & demonstration of effect of bLF Vancomycin resistant *Enterococcus faecium* (VRE). The positive synergistic effect of LF along with antibiotics tested is marked by rectangles (in red) in Figure 3, Figure 4, Figure 5, Figure 6, Figure 7, Figure 8 and Figure 9 and summarised in Table 1. 

To specify some of the phenotypic effects of LF in synergism with antibiotics: the isolates producing ESBL (extended-spectrum β-lactamases) had turned non-ESBL; quinolone resistant isolates had turned susceptible; MRSA (methicillin resistant *S. aureus*) had turned MSSA (Methicillin susceptible) and vancomycin resistant Enterococci (VRE) had turned susceptible. The results were found to be totally reproducible each time. 

All the total 147 isolates were tested against bLF and the result was remarkable in turning all the 147 varied resistant isolates into susceptible. The analysis of the isolates exposed to bLF in synergism with antibiotics did not demostrate any observable change in the molecular basis (genes coding for broad-spectrum β-lactamases) of drug resistance. There seems to be changes only in the gene expression after the exposure to LF, which cannot be declared without the experimental support, such as qPCR or transcriptomics.

The effect of natural LF on the ability of biofilm formation of pathogens studied using a microculture protocol against pathogens of interest was denostrable. LF had remarkably inhibited the biofilm production. The results were reproducible. 

## 4. Discussion

The Multidrug-resistant (MDR) bacterial infections, particularly those caused by Gram-negative pathogens, have arisen as one of the world’s utmost health issues. The development of novel antibiotics against MDR GNB has declined over the last half century. There is research in progress regarding the therapeutic strategies that could be engaged in conjunction with antibiotics which could extend the life span of these life-saving drugs [23]. 

In our study, LF on its own did not display any effect on the studied pathogens. However, the synergism effect of LF with antimicrobial compounds (antibiotics) after optimising the MIC (minimum inhibitory concentration) based tests were found to be remarkable. To specify some of the phenotypic effects of LF in synergism with antibiotics: the isolates producing ESBL (extended-spectrum β-lactamases) had turned non-ESBL; quinolone resistant isolates had turned susceptible; MRSA (methicillin resistant *S. aureus*) had turned MSSA (Methicillin susceptible) and vancomycin resistant Enterococci (VRE) had turned susceptible. The results were found to be totally reproducible each time. 

In our study, the molecular analysis of the isolates exposed to bLF in synergism with antibiotics were carried out to determine any change in the molecular basis of antimicrobial resistance. There was no observable change in the molecular basis (genes coding for broad-spectrum β-lactamases) of drug resistance. There seems to be only changes in the gene expression after the exposure to LF compounds, which cannot be declared without the experimental support, such as qPCR or transcriptomics.

In our study, the effects of LF on biofilm formation were optimized by microculture protocol. LF on its own had a significant effect of inhibiting the biofilm production.

According to the original findings of LF, first antimicrobial properties discovered was sequestering of iron from bacterial pathogens to inhibit bacteria growth [24]. But later the research findings confirmed that LF is also able to destroy bacterial pathogens by an iron-independent mechanism, by direct interaction with the bacterial cell surface [25].

LF has antimicrobial activity against a range of various bacterial pathogens, through host cell invasion strategies, iron sequestration, targeting of bacterial virulence mechanisms and membrane destabilization. In general, the antimicrobial mode of action of LF is hugely dependent on the conditions of experiments [26].

In a study by Bhimani et al., 1999 [27], cLf revealed substantial inhibitory effect versus *E. coli* followed by *P. aeruginosa*, *S. agalactiae* and *S. aureus*. Biofilm formation renders the bacteria such as *P. aeruginosa* highly resistant against antimicrobial treatment and host cell defense mechanisms [28]. The bacterial strains need increased levels of iron for biofilm formation. Consequently, due to Lf’s role as iron chelator has been postulated to successfully inhibit the formation of biofilms via iron sequestration [29]. 

Kutila et al., 2003 [29], explored the antibacterial effect of bLF against udder pathogens. The best inhibitory activity was observed against *E. coli* and *P. aeruginosa*. The study showed variable response to LF against *S. aureus* and CNS isolates. The study confirmed that bLf is antibacterial against the major pathogens. Whereas, Nonnecke & Smith, 1984 [30], testified only bacteriostatic effect of bLF against *E. coli* and *K. pneumoniae*. Dionysius et al., 1993 [31], demonstrated that Lf (1.0 mg/mL) inhibited growth of [19 isolates] ETEC (enterotoxigenic *E. coli*) cultured from porcine enteritis. 

## 5. Conclusions

LF has been the focus of more intense research. Due to its unique antimicrobial, immunomodulatory, and even antineoplastic properties, LF seems to have great potential in practical medicine. Nevertheless, much research and many experiments still need to be carried out in order to obtain a better understanding of its activity and interactions and to enable the full and safe utilization of this glycoprotein. The outcome of the study suggests the strategy of using LF in conjunction with conventional antibiotics, primarily by having direct effect on the pathogen besides probable role of enhancing the immune system of the host without incurring prohibitive toxicity which might prove to be beneficial in designing alternative anti-infective therapeutic agents. This study is supportive of alternative conjunction based treatment aspects of infections caused by antibiotic resistant bacterial pathogens.

The results of this study point towards the possible use of LF as an adjunct to appropriate conventional antibiotics and helps in developing alternate strategies to combat bacterial infections caused by the drug resistant pathogens. 

The LF ingredients prove to be highly useful in alternative medicine and newer area of further research for scientists involved in biopharmaceuticals. The study provides us with solution for the emerging and spreading antimicrobial resistant bacterial pathogens. It provides useful data on the antibacterial potential of the combination of LF with antibiotics against drug resistant pathogens.

This study forms a pilot study suggestive of more detailed research project to be carried out to help the implementation of the concept generated. 

The study mainly helps the health authorities to design plans to curb the spread of infections causing drug resistant bacteria. The information of escalating antimicrobial resistance could help the pharmaceutical companies to aim for newer LF based synergistic antimicrobial agents. 

## Figures and Tables

**Table 1:** Overall results of the isolates tested against the bovine lactoferrin (bLF).

Sl. No.	Clinical Isolates Tested	bLF
1	Methicillin resistant *Staphylococcus aureus* (MRSA)—30 isolates	All 30 tested. All positive for LF action.
2	Methicillin resistant Coagulase negative *Staphylococcus*—30 isolates.	All 30 tested. All positive for LF action.
3	Extended-spectrum beta-lactamase (ESBL) producing *Enterobacteriaciae*—40 isolates.	All 40 tested. All positive for LF action.
4	Fluoroquinolone resistant Gram negative pathogens—30 isolates.	All 30 tested. All positive for LF action.
5	Multidrug resistant *Pseudomonas* species—05 isolates.	All 05 tested. All positive for LF action.
6	Carbapenem resistant Gram negative pathogens—05 isolates.	All 05 tested. All positive for LF action.
7	AmpC β-lactamase producing Gram negative pathogens—05 isolates.	All 05 tested. All positive for LF action.
8	Vancomycin resistant Enterococci (VRE)—02 isolates	All 02 tested. All positive for LF action.
	Total isolates tested	*n* = 147

## Data Availability

All datasets generated or analyzed during this study are included in the manuscript.
